# The Residential Population Generator (RPGen): Parameterization of Residential, Demographic, and Physiological Data to Model Intraindividual Exposure, Dose, and Risk

**DOI:** 10.3390/toxics9110303

**Published:** 2021-11-12

**Authors:** Alexander East, Daniel Dawson, Graham Glen, Kristin Isaacs, Kathie Dionisio, Paul S. Price, Elaine A. Cohen Hubal, Daniel A. Vallero

**Affiliations:** 1 Center for Computational Toxicology and Exposure, U.S. Environmental Protection Agency, Office of Research and Development, Research Triangle Park, NC 27709, USA; east.alexander@epa.gov (A.E.); dawson.daniel@epa.gov (D.D.); isaacs.kristin@epa.gov (K.I.); dionisio.kathie@epa.gov (K.D.); price.pauls@epa.gov (P.S.P.); 2 Oak Ridge Associated Universities, Oak Ridge, TN 37830, USA; 3 ICF, Inc., Durham, NC 27713, USA; graham.glen@icf.com; 4 Center for Public Health and Environmental Assessment, U.S. Environmental Protection Agency, Office of Research and Development, Research Triangle Park, NC 27709, USA; hubal.elaine@epa.gov

**Keywords:** exposure assessment, National Health and Nutrition Examination Survey (NHANES), exposome, probabilistic exposure model, vulnerable populations, Combined Human Exposure Model (CHEM), Residential Population Generator (RPGen)

## Abstract

Exposure to chemicals is influenced by associations between the individual’s location and activities as well as demographic and physiological characteristics. Currently, many exposure models simulate individuals by drawing distributions from population-level data or use exposure factors for single individuals. The Residential Population Generator (RPGen) binds US surveys of individuals and households and combines the population with physiological characteristics to create a synthetic population. In general, the model must be supported by internal consistency; i.e., values that could have come from a single individual. In addition, intraindividual variation must be representative of the variation present in the modeled population. This is performed by linking individuals and similar households across income, location, family type, and house type. Physiological data are generated by linking census data to National Health and Nutrition Examination Survey data with a model of interindividual variation of parameters used in toxicokinetic modeling. The final modeled population data parameters include characteristics of the individual’s community (region, state, urban or rural), residence (size of property, size of home, number of rooms), demographics (age, ethnicity, income, gender), and physiology (body weight, skin surface area, breathing rate, cardiac output, blood volume, and volumes for body compartments and organs). RPGen output is used to support user-developed chemical exposure models that estimate intraindividual exposure in a desired population. By creating profiles and characteristics that determine exposure, synthetic populations produced by RPGen increases the ability of modelers to identify subgroups potentially vulnerable to chemical exposures. To demonstrate application, RPGen is used to estimate exposure to Toluene in an exposure modeling case example.

## 1. Introduction

Reliable exposure information and predictions are essential to risk assessments and other tools used to support public and environmental health decisions. The likelihood and extent of risk are governed by a person’s exposome [1,2,3,4,5], which reflects a person’s unique biological makeup, behavior, and environmental exposures.

The National Research Council (NRC) has provided guidance on ways to assess risks of chemicals, including reports that highlighted the disparity between the rate of deployment of new anthropogenic chemicals and assessment of their potential risks to public health [6,7]. Central to these evolving recommendations has been the replacement of animal-based characterization of chemical hazard and extrapolation to human health with high-throughput in vitro tests, in silico models, and evaluations of efficacy at the human population level. Noting that risk is a function of both hazard and exposure, the NRC added recommendations for advancing the risk-based science that underpins environmental and human health decision making [7,8].

Throughout most of its half-century of existence, the risk assessment community has viewed pollutants from a “far-field” perspective. That is, a substance is released into the environment, and exposure occurs later and at some distance from the source. In a far-field scenario, exposure concentration is a function of the transport and transformation of the chemical compound in time and space [9,10,11,12,13]. However, increasingly, the US and other nations have recognized that for some chemicals and uses, far-field exposure accounts for much less of the potential exposure likelihood and concentration than do exposures in microenvironments [14,15]; e.g., time spent in residences. The emissions and contact with consumer products, articles, and building materials can account for most of these “near-field” exposures [16,17,18,19,20,21]. As evidence, the Toxic Substance Control Act and other laws have authorized greater attention to “near-field” exposure scenarios [22]. A near-field consumer exposure scenario is one in which exposure to a chemical occurs by humans who use a product or by bystanders who are in the vicinity of the product use. The exposure pathways particularly important in near-field scenarios are the inhalation of chemicals released from products indoors, dermal uptake of substances from consumer products and articles, and oral ingestion of compounds in food and beverages, as well as incidental ingestion; i.e., oral intake of compounds by hand-to-mouth activities, which is known as non-dietary ingestion [15,23]. The transition to an increased emphasis on near-field exposure scenarios amplifies the need for reliable predictions of human activities in time and space. In particular, modeling near-field exposures scenarios requires improved capabilities to associate individuals with the residential environments in which exposures occur. This recent emphasis is also enhancing the understanding of prioritization between near-field and far-field exposure scenarios in the composition of total exposure estimates [10].

Near-field exposures are strongly influenced by individual behavior and circumstances. For example, the use of a particular product in a residence may be dependent upon the sex, age, and other demographic characteristics of a person. Thus, an important challenge of estimating near-field exposure in a population is characterizing the underlying variability of that population. A common approach to capture such variation in exposure modeling is to sample individuals from a population of interest, based on the distribution of key parameters, including exposure-related behaviors [15,24,25,26]. For example, a parameter describing the frequency that particular individuals (e.g., adult males) engage in a particular activity (e.g., bathing) can be sampled from a distribution, influencing exposure to the products (e.g., soap, shampoo) associated with that activity [21]. For such modeling efforts, data describing exposure-related behaviors have been collected and summarized [27] and are also estimated in the EPA Exposures Factors Handbook [28]. While these sources present data on the distributions of values between individuals, such models often do not capture the correlations between demographics, physiology, residence, and exposure factors. In addition, since data on interindividual variation are often collected from a variety of sources, it is not clear if exposure estimates are representative of any specific population. For example, the Stochastic Human Exposure Model (SHEDS-HT) [24] utilizes demographic information to associate exposure behaviors based on age and gender, but it integrates consumer product use data collected in aggregate surveys of both North American and European populations to make exposure estimates. However, a population used to capture intraindividual variation must be internally consistent (i.e., reflect data that could come from a single individual), and the variation in the sets across individuals should be representative of variation in the modeled population. For example, physiological characteristics such as the breathing rates and body weights of adults are expected to be positively correlated (internally consistent), and the probability of owning an automobile may differ for individuals living in rural lower-income households compared to individuals living in urban high-income residences (consistent across a population), both of which inform exposure to chemicals.

Models used to predict exposure to chemical ingredients in products are based on chemical properties, human activities, and product use [24]. By linking exposure estimates to dose-dependent toxicity, exposure risks are estimated. As is typical for any modeling process, various data inputs are required. Often, however, some of the more significant areas of data paucity for exposure modeling lie in the characterization of residential and populations aspects of exposure. For example, Huang and colleagues [29] recently demonstrated that additional data on exposure factors are needed for population-scale, near-field exposure assessments due to substantial variability in both the quantity (i.e., dose) and context of exposures. The latter includes aspects that influence near-field exposure, including within-building air flow, surface areas, the composition of articles, and the presence of interior appliances. Thus, reliable exposure estimates and predictions need descriptions of the physical structure, home contents, and other exposure factors. To this end, the U.S. EPA developed the Residential Population Generator (RPGen) within the Combined Human Exposure Model (CHEM) framework [24,30]. This framework is designed within the federal government’s open source policy [31], and it aims to model aggregate, longitudinal exposure to chemicals of interest using the open source R software [32]. In addition, all the modules within CHEM, including RPGen, were built to be interoperative. Therefore, RPGen exists outside of any standalone model and can be imported by other models, tools, and dashboards.

## 2. Methods

RPGen was initially developed in 2016 in conjunction with two other modules in CHEM: Product Use Scheduler (PUS), and Source-to-Dose (S2D). PUS accepts the generated population from RPGen and creates product use diaries depending on the characteristics of the individual and household. Then, S2D uses product formulations and the use diaries to create 1-year longitudinal estimates of exposure from chemicals in consumer products and down-the-drain release of chemicals. The development and presentation [33] of RPGen is ongoing, and updates to ease-of-use, transparency, and new data refreshes are catalogued on GitHub in the Supplementary Materials.

**Figure 1:**
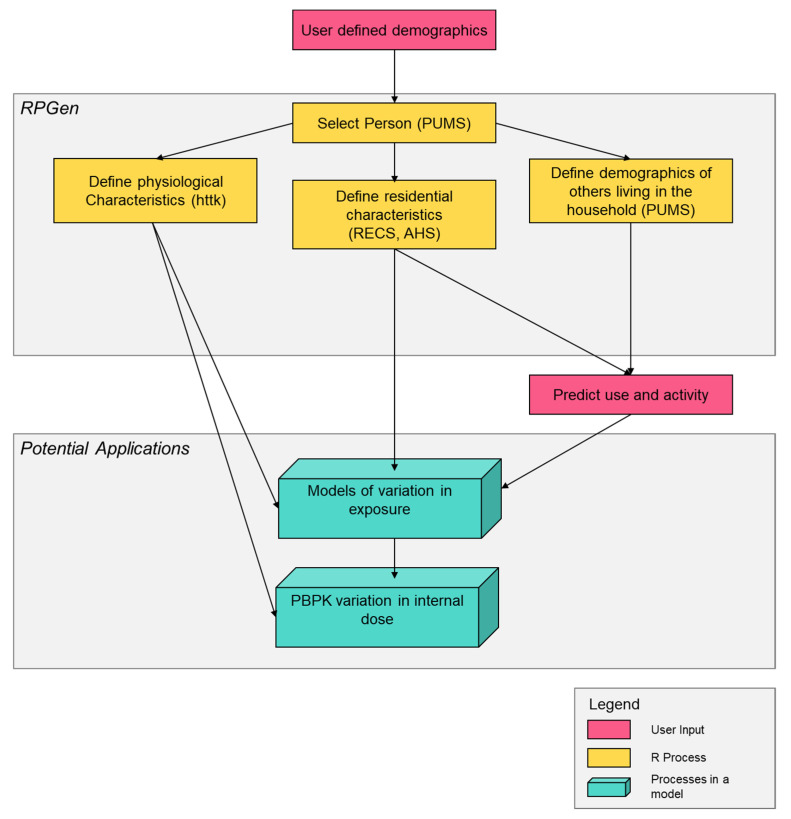
Role and internal process of RPGen in exposure and dose models. Note: Figure references the Public Use Microdata Survey (PUMS), the Residential Energy Consumption Survey (RECS), and the American Housing Survey (AHS).

RPGen is a collection of scripts and functions that assembles a synthetic, demographically representative population, which is suitable for estimating exposures to chemicals of interest and provides descriptions of inputs for use in models of exposure and dose. For example, RPGen supports models of far-field exposures by describing the variation in residential and personal characteristics by region and community type (urban/rural) and supports models of near-field exposures that occur in and around the home by defining the variation in model inputs that describe the residence and presence of certain sources of chemical exposures (e.g., garden, appliance, pool, car, etc.). In addition, RPGen provides individual physiological variables to support physiologically-based pharmacokinetic (PBPK) models of internal dose. In brief, RPGen creates synthetic populations useful for exposure modeling by sampling individuals included in the publicly available US population and housing surveys. See Figure 1 for a conceptual overview of this process.

RPGen is run by creating an input text file or entering the parameters directly into the R console. Possible inputs are included in Table 1 below. RPGen populations are generated based on parameters defining the individuals, such as min.age and max.age, and location, such as states. The module output is a csv file, in which each row contains information for one individual, and each column represents one of 126 variables associated with the individual. RPGen handles blank inputs with inclusive defaults. For example, in a run with states and regions left blank, RPGen would use all contiguous US Federal Information Processing Standards (FIPS) codes. For more detailed information about downloading, running, and using RPGen, the RPGen Technical Manual is available in the GitHub repository, which is provided in the Supplementary Materials.

No single survey includes all the demographic, residential, and physiological information needed for assessing individual chemical exposures. Thus, records from three surveys are combined to create a synthetic population: The Residential Energy Consumption Survey [34], the American Housing Survey (AHS) [35], and the 5-year versions of both the Housing and Person Public Use Microdata Surveys (PUMS) [36] (Table 2). Exposure-relevant housing information is provided by AHS and RECS, whereas PUMS is a sample of the US census that contains individual-level records on exposure-relevant demographic factors, including gender, age, and ethnicity. Variables from AHS are characteristics of households (bathrooms, bedrooms, water source), and variables from RECS are features of the household that pertain to energy use (presence of a washing machine, number of cooktops, fuel used). This information informs household exposures to chemicals from cooking and cleaning activities. Some limited housing data are also imported from PUMS and used to link individual records to the housing data found in AHS and RECS using the process described in Section 2.3 below. The current version of RPGen uses the most recent data available from PUMS, AHS, and RECS (Table 2). New PUMS data are released annually, AHS data publication occurs biennially, and RECS data are published every four years.

Physiological data are estimated per individual using the R packagehttk [37,38], which generates inputs for pharmacokinetic models using data from the National Health and Nutrition Examination Survey (NHANES) dataset. RPGen does not import NHANES data, instead calling httk to estimate physiologies using PUMS race, ethnicity, and age data. RPGen also applies stochastic variation to the httk output to create unique physiologies for similar individuals. In contrast to survey data from RECS, AHS, and PUMS, the physiological variables are sampled from distributions.

To merge census and housing records between the three datasets, a common variable pool was derived from a suite of 5 shared categorical variables. These variables included setting, region, house type, family type, and income category. One designation from each column is possible for each individual or household, resulting in 288 potential assignments in the pool. RPGen selects individuals and households with matching pool values in each survey to generate the synthetic population. Table 3 contains the variables that create unique pool values that were used to link datasets. The pool variable (1–288) associated with each member of an assembled population is also included in the RPGen output.

### 2.1. Setting

Both RECS and AHS include variables for micropolitan and metropolitan statistical areas, which were issued by the Office of Management and Budget (OMB) in 2010 [39]. Metropolitan areas have at least one urbanized area containing 50,000 or more individuals and micropolitan areas contain a cluster of at least 10,000 but less than 50,000 individuals and exist adjacent to a metropolitan statistical area. Other more rural delineations are provided in the OMB’s standards but are not coded equally into AHS and RECS. As such, all metropolitan statistical areas are coded as urban, and all other areas are considered rural.

PUMS does not currently provide an “urban/rural” statistical area designation, but instead uses Public Use Microdata Areas (PUMAs) that contain approximately 100,000 people and consist of complete census tracts. To link the urban/rural variable in AHS/RECS with corresponding information in PUMS, population densities for each PUMA were determined. The threshold for Urban PUMASs was set at >130 people per km^2^. This was equivalent to the population density of Chapel Hill, North Carolina in 2016, which is a relatively small city (approximately 70,000 people) largely surrounded by agricultural and forested landcover. Under this threshold, a PUMA was classified as rural. Each input dataset contains a region specification (North, South, Midwest, West), so the urban/rural setting designation is combined with region to create eight geographic location possibilities.

### 2.2. Region

**Figure 2:**
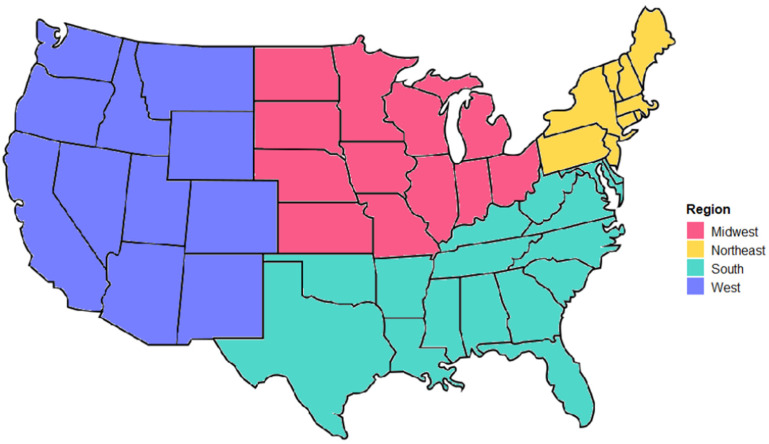
Regions of the United States.

Both AHS and PUMS contain PUMA codes. However, RECS only contains a metropolitan, micropolitan, or neither designation using Census Micropolitan Statistical Areas (CMBAs). This results in a loss of location granularity for households: as households drawn from RECS can only be determined by region and CMBA, specificity at a county or state level is not possible for households. Figure 2 shows the four regions available from which a household can be designated as urban or rural. Location granularity for individuals generated in RPGen is at the PUMA level, as the person is generated first within a state and then assigned a household using the pool variable, but households are limited to eight possible locations used to match individuals and residences.

### 2.3. Income

Income was split evenly into three bins: high, middle, and lower, which create the income category variable. To account for location-bound variation in purchasing power, income category was calculated by first splitting households into one of the eight possible locations (e.g., urban, Midwest), and then calculating the income category. The income category variable, inccat, is included in RPGen’s output, and a household income variable income is carried from AHS to the final output.

### 2.4. House Type

House types were condensed into three common categories: standalone, multi-structure, and other housing units. Houses and attached houses are considered standalone, while apartments and condominiums are considered multi-structure units. The other classification includes mobile homes and boats. PUMS has housing and person data for individuals in group quarters, such as military bases, prisons, and homeless shelters. Since these classifications are not included in AHS or RECS, RPGen excludes these house types. Houses reported as empty in AHS were also discarded.

### 2.5. Family Category

Families are categorized into 4 bins: single adults living alone, adults with no children, single adults with children, and adults with children. In RPGen, adults are considered to be 18 years of age and above. Although there were no households without adults recorded in the PUMS 2014–2018 dataset, RPGen filters any households without at least one adult. Individuals from outside of the contiguous US are also discarded, as RECS and AHS only surveyed contiguous US households.

### 2.6. Population Assembly

Once pool is assigned for records in all three datasets, RPGen assembles a synthetic population that is statistically representative of demographic patterns in the data based on matching pool values. The 2014–2018 PUMS dataset consists of 15,094,428 randomly sampled census records and thus serves as the basis of the sampling and assembly process. Each individual in the PUMS dataset is assigned a statistical sampling weight, which reflects the estimated number of similar individuals (based on a suite of demographic characteristics) that live in the US according to the census data (US Census Bureau, 2020). To utilize these sampling weights for sampling individuals from PUMS, RPGen uses a two-step process similar to that developed for use in the USEPA Air Pollutants Exposure Model [40]. First, a vector of random numbers between 0 and 1 is generated for the desired number of samples. Next, the statistical sampling weights in the PUMS dataset are assembled into a cumulatively summed vector that is divided by the maximum value of that vector, resulting in a final vector that ranges from 0 to 1. The interval sizes between values of this vector reflect the relative statistical sampling weights of each person in the PUMS dataset. The random numbers are paired with the intervals in which they fall in the PUMS weight vector. Then, the matched intervals are selected to become members of RPGen. Since individuals with larger statistical weights result in proportionately larger intervals than those with smaller weights, it is more likely that a random value will fall into them. Thus, individuals are more or less likely to be included in RPGen based on their weight in PUMS.

Following the selection of individuals from PUMS into RPGen, housing characteristics are sequentially matched to each person based on their pool value using a similar process. First, all the values in AHS or RECS that have matching pool values to an individual are identified. Then, using statistical sampling weights specific to both RECS and AHS, the same two-step sampling process described above is employed. The completed output is a synthetic population of people within households that reflect real underlying demographic variation, and it maintains the correlation structure between physiological and demographic characteristics. In addition, the above approach is coupled with functions specifying control over random-seed generation. This allows users to ensure the reproducibility of sampled populations as well as facilitating sensitivity and uncertainty analyses of the different datasets.

The PUMS record defines the age, gender, race, and ethnicity of the individual. RPGen subsequently calls the R package httk, which generates physiological variables for each individual from PUMS using ethnicity and age. As discussed by Pearce and colleagues [37], httk provides data sets of internally consistent values for a set of individuals. The package generates the relevant data from NHANES used to calibrate equations that predict internally consistent measurements of physical (height, weight) and physiological measurements (e.g., cardiac output, organ size, serum creatinine, and hematocrit) based on age, gender, and ethnicity that vary consistently with the US population [41]. RPGen calls httk such that some individuals with a matching age and ethnicity will have matching physiological output. Therefore, RPGen introduces variation by the jittering of variables, which includes limits on height and weight of 225 cm and 160 kg. Although individuals exist that exceed these values, the randomization elements added to RPGen are curtailed at these extremes.

### 2.7. Role of RPGen

RPGen was written to assemble sample populations that represent underlying demographic patterns present in the US population for models of exposure. Most of the variables in RPGen are categorical—determining whether a household or family has a washing machine, for instance, informs use and exposure to related chemicals. Intraindividual exposure from individuals of the same residence allows for increased granularity when modeling the exposome. Furthermore, calculating exposures that may derive from other household members (particularly important for children) maintains correlation structures between people and aspects of households that influence exposure. To demonstrate the breadth of RPGen, two example runs of the module with 5000 people representing all genders, ethnicities, states, and regions were performed. The first file has an individual age range of 0–99 and the second ranges from 0 to 21. RPGen output files for each run are available in the Supplementary Materials.

### 2.8. Toluene Exposure for Homeowners and Non-Homeowners Case Example

To demonstrate the application of RPGen in a model, a case example for exposure to toluene from household products was performed. In RPGen, the variable kownrent describes if the individual is in a rented household, staying without rent, or owns a household. In CHEM, the model component called the Product Use Scheduler (PUS) accepts a population from RPGen as input and creates use diaries depending on the characteristics of the individual and household. Therefore, product use categories often associated with owning a household were set to be assigned only to owners of households in the RPGen population. This suite of categories includes deep cleaners, stainers, caulking, and sealant. A complete breakdown of product use categories and products containing toluene in CHEM is available in the Supplementary Materials.

The guiding theory behind these rules is that owners are exposed to a unique subset of products because of actions associated with a home. It is implied in PUS that individuals that do not own a household do not partake in deep cleaning and repair projects that owners would.

Once PUS has determined product use diaries for each individual and co-inhabitants in RPGen, the diaries are passed to the Source-to-Dose (S2D) module, which estimates exposure and down-the-drain using product formulations and route-specific exposure equations. In this manuscript, the mean daily total exposure, or sum of direct and indirect exposure across inhalation, ingestion, and dermal pathways over a year is compared with SHEDS-HT, which is a one-day cross-sectional model. The population in SHEDS-HT (v0.1.8) [42] was generated using default consumer product use patterns. In SHEDS-HT, these default patterns (e.g., mass used, population prevalence, and frequency) are explicitly defined by age and gender cohort based on the available literature and reasonable assumptions. However, these cohort differences are usually quite crude, as few have enough data to allow for refined stratification; most differences are quantified for males versus females (e.g., for cosmetics) or for adults versus children (e.g., for direct use of home maintenance or auto products).The quantitative weight fraction data used to generate the chemical-specific SHEDS inputs for each product category were identical to those used in the CHEM run (reformatted for the SHEDS format), which is the CHEM default data derived from the 2019 release of EPA’s Chemicals and Products Database (CPDat). All other SHEDS input files were the default versions.

Given the increased population granularity in RPGen, the CHEM run is split into exposures for owners (own the household) and non-owners (rent or stay without rent), which is a capability not included in SHEDS-HT. Given long processing times, a run of 1000 individuals from RPGen was used in CHEM compared to 10,000 in SHED-HT. Default population characteristics were used: no restrictions were placed on location, ethnicity, or age of the individuals in RPGen. The same consumer products lists and product formulations were used for both SHEDS-HT and CHEM. In addition, to enhance the case example, RPGen was also ran with only the Product Use Categories (PUCS) [24] pertaining to home ownership to evaluate the contribution of home ownership to total exposure estimates. In total, three runs were performed: CHEM, SHEDS-HT, and CHEM with homeowner-only PUCS. Further analyses were performed by subsetting the output of the models.

## 3. Results

### 3.1. RPGen Capabilities

**Figure 3:**
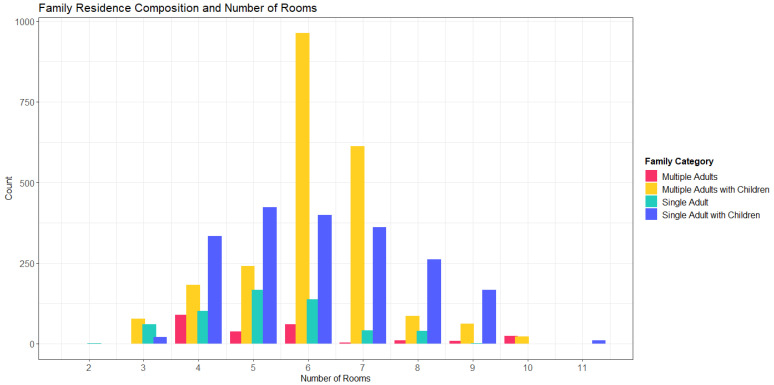
Family category histogram, *n* = 5000.

RPGen output variables assist in the prediction of whether individuals will use certain types of consumer products. For example, if an individual resides in a household that has a swimming pool, the occupants are at a greater likelihood of exposure to pool cleaners than individuals living in a household without a pool. Output variables inform exposure by building the general external environment that informs behavior and habits. Additionally, the assembly of households allows for the modeling of interindividual exposure or exposure between people. The family category, used to link survey datasets, describes the composition of a household. As presented in Table 3, the family category has four possibilities: single adults living alone, adults with no children, single adults with children, and multiple adults with children. Figure 3, generated from a sample run of all ages, demonstrates how the family category is distributed among the number of rooms in a household. In this run, adults with children tend to have homes with more rooms than adults without children, and single adults occupy households with fewer rooms. Furthermore, there are more households with children than without. Understanding household composition, in conjunction with residence size, often characterizes the fugacity of chemicals in larger spaces. Furthermore, demographics of occupants inform habits and product use in the exposome: households with and without children and differing numbers of adults apply different products and thus have different exposure profiles, or exposomes. By capturing interindividual variation, RPGen reflects realistic underlying exposure considerations and generates potentially useful parameters for models.

**Figure 4:**
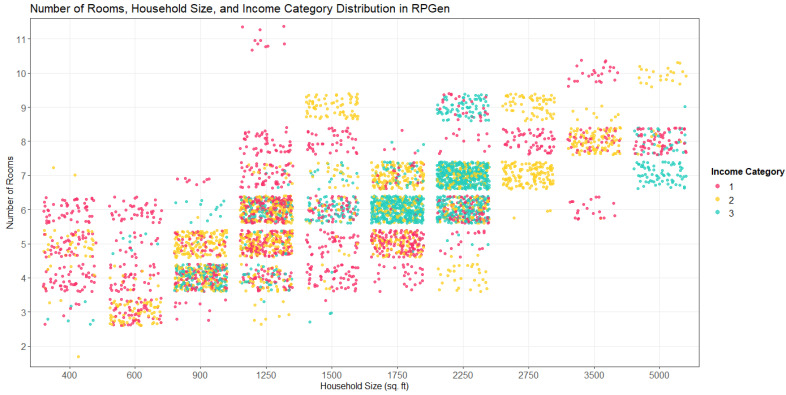
Households and income category, *n* = 5000, jittered.

In addition, when the number of rooms in a household is considered together with household size and income category, we find that there is an overall positive relationship between all three variables (Figure 4). The density of the jittered distribution also demonstrates the prevalence of each household characterization: there are more residences at 1250 sq. ft and 5 rooms than 5000 sq. ft and 12 rooms. Additional determinants of air flow, such as the number of ceiling fans, number of windows, and high ceilings are also included in RPGen to provide inputs to air exchange rate models, as air flow within a household will greatly influence indoor air chemical concentrations [43]. Furthermore, the inclusion of income level and whether households have children or not likely influence both the nature and extent of product purchase and use. When coupled with an exposure model, these simple metrics may influence exposure estimates, but additional demographics, such as income, may also indicate particularly at-risk subpopulations. Figure 3 and Figure 4 are generated from the same sample run of RPGen described in Section 2.7.

**Figure 5:**
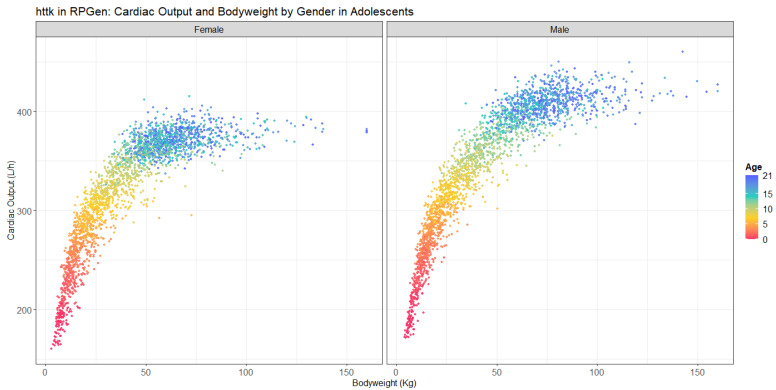
Adolescent (21 and under) cardiac output in males and females, *n* = 5000.

In addition to exposure factors associated with housing, interindividual physiology critically influences exposure and subsequent toxicity. For example, exposure-relevant physiological factors change quickly in adolescents, varying by age and gender [44]. By leveraging the httk package, RPGen captures a suite of physiological information necessary for estimating physiologically mediated exposure. The relationship between breathing rate, an important physiologic important parameter in estimating exposure through inhalation, with body weight is shown to vary by sex and age (Figure 5). The following nationwide sample run was generated with max.age set to 21. The randomization and caps of the physiological variables mentioned in Section 2.6 are visible: two generated males and one female have been assigned the cap of 160 kg. Additionally, there is a visible positive correlation between cardiac output for males and females through adolescence, with grown males often having greater bodyweights and cardiac outputs at similar ages.

### 3.2. Toluene Case Study Results

Residential exposure to toluene is compared across homeowners and non-owners in CHEM and SHEDS-HT. In 659 individuals within owned homes in CHEM, 517 were exposed to toluene: 78.5%. Across the 341 non-owners, 102, or 29.9% registered an exposure to toluene. In SHEDS-HT, 9437, or 94.4% of 10,000 were exposed.

**Figure 6:**
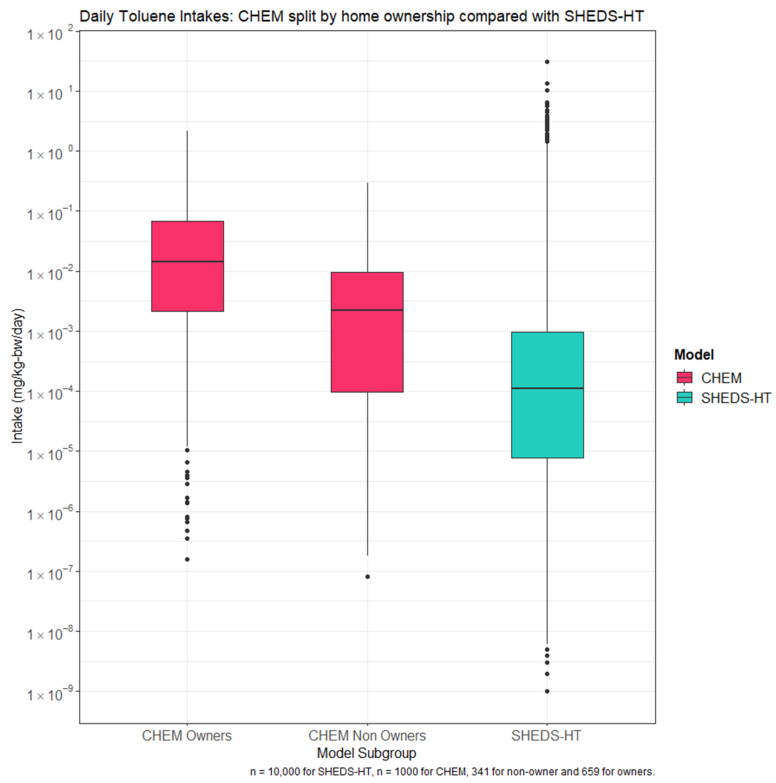
Daily toluene intakes across CHEM owners, non-owners, and SHEDS-HT.

The means of each model subset, including households that did not register exposures, are 0.023 mg/kg-bw/day for SHEDS-HT, 0.067 mg/kg-bw/day for CHEM Owners, and 0.0041 mg/kg-bw/day for CHEM non-owners. The standard distributions are 0.404 for SHEDS-HT, 0.165 for CHEM owners, and 0.234 for CHEM non-owners. The distribution of all three curves is lognormal. The disparity in these model estimates shows that different outcomes are estimated by using a population with more granular descriptors. Figure 6 contains boxplots for each subset. Given the larger number of samples, SHEDS-HT has a greater spread than CHEM outputs. Additionally, individuals without exposure are not expressed in the boxplot, so a greater disparity between results is observed in the figure than comparison of the means.

An additional run for product use categories (PUCS) only pertaining to home ownership was also performed. The mean daily intake of these households, among households with registered exposures, was 0.71 mg/kg-bw/day, compared to 0.78 mg/kg-bw/day for owners with all PUCS in the previous all-inclusive run. Given this, approximately 91% of a homeowner’s toluene exposure is from PUCS pertaining to owning a home. However, non-homeowners have a mean exposure of 0.30 mg/kg-bw/day, so the relative contribution to total exposure is not characterized by the delta of the all-inclusive homeowner run and the homeowner PUCs only run. This is because PUS assigns other activities that result in exposures for non-homeowners when homeowners would be performing homeowner unique tasks. Rather, CHEM provides an estimated baseline of 0.30 mg/kg-bw/day for non-homeowners, which increases to 0.78 mg/kg-bw/day for homeowners, of which 91% is from ‘homeowner’ PUCS. More summary statistics are available in the Supplementary Materials.

## 4. Discussion

### 4.1. Limitations

#### 4.1.1. Geographic Resolution

As discussed, residences in RPGen are matched by eight location types: one of four regions are selected, and setting is assigned as either urban or rural. This is due to the lack of resolution in RECS: both AHS and PUMS contain PUMAs, but household matching can only be determined by the location data provided in RECS. However, because individual population profiles are from PUMS, the people generated from RPGen have greater geographic resolution, and they are location bound by PUMAs, unlike the generated residences, which are matched by region.

#### 4.1.2. Exposure Pathways

Since RPGen was originally designed to operate within the CHEM framework, the physiological parameters and exposure factors reflect a focus on modeling residential exposure to consumer products. For example, the physiological parameters inhalation rate (CO) and body surface area (BSA_adj) support inhalation and dermal modeling exposure to products used and applied within a household. However, to characterize exposures to chemicals by all pathways, including dietary and water ingestion, additional models and data are required [45]. Future efforts are underway to add data to RPGen’s physiological outputs that would support the generation of populations to assess exposures associated with soil, dust, water, and diet.

#### 4.1.3. Other Data Limitations

Income category is coded as 1, 2, or 3, with 1 representing the highest income category and 3 as the lowest, with each category containing the same number of individuals. However, these bins could mischaracterize the distribution of wealth in US households [46]. Additionally, RPGen does not include individuals living in prisons, shelters, and military bases, as there are no residence data available in RECS or AHS. Therefore, alternative population generation approaches would likely be more appropriate in these situations. Furthermore, due to the small sample sizes of 5686 and 57,972 respectively, RECS and AHS input files do not contain households for every possible pool. This oftentimes occurs in the case of a rural, high-income apartment. Therefore, an evaluation of assembled pools may be necessary to assess RPGen’s application to particular questions. For more information on the pool variability, the RPGen Technical Manual is available in the Supplementary Materials. Additionally, RPGen provides no adjustments regarding time. For long-term models involving diet and pharmacokinetics, the population generated by RPGen would be expected to gradually change, introducing uncertainty into the model. RPGen is constructed to accept new data from RECS, AHS, and PUMS as released, but it does not have a strict update schedule.

### 4.2. Case Study Discussion

CHEM, and therefore RPGen, is best used for estimates of aggregate exposure across products. For single-use products or chemicals that are present only in one product, characteristics of the household do not enhance the exposure predictions. However, within CHEM, toluene is in 229 products, 200 of which are influenced by the home ownership rules. Four other chemical exposures—benzyl butyl pthalate, diethylene glycol, dibutyl pthalate, and naphthalene—were also simulated CHEM. However, these chemicals had relatively low exposure counts over 1000 individuals and featured more frequently in products not associated with the home ownership rules. Given that these chemicals are found less broadly across PUCS and products, relationships between exposure outcomes and homeownership are less clear than those for toluene. The results of these runs are available in the Supplementary Materials. An additional concern of agent-based modeling is the extended runtime. For five chemicals, 1000 households in CHEM exceed 100 h runtime for a machine with 8 GB RAM, while 10,000 estimates in SHEDS-HT estimates were generated in under 5 min. This is why different numbers of individuals were used for the RPGen and SHEDS-HT runs and is a consideration when building and appending models to CHEM.

### 4.3. Other Exposure Models

Computational models of exposure and dose are fundamental to risk assessment and are only as effective as the least resolved component of the model [47]. To meet this end, exposure model capabilities and frameworks continue to expand dramatically [48]. Similar previous efforts to capture such population variability to model exposure include the aforementioned SHEDS-HT and PopGen [21,49]. PopGen creates individuals for PBPK models and was improved in the httk package with more detailed NHANES sampling and newer data [41]. Meanwhile, in the field of epidemiology, the Framework for Reconstructing Epidemic Dynamics (FRED) model uses PUMS data in an agent-based modeling approach that captures demographic and geographic heterogeneities of the population, including realistic household, school, and workplace social networks [50]. RPGen builds on these other population generators by tying together specifics of the household and physiology at the individual level. RPGen may also be applied to models of inhalation, as descriptions of the household can be used as the basis for agent-based models. For example, exposure models that measure exposure outcomes between time indoors and time outdoors may be influenced by the presence of a vehicle, the structure of the household, number of windows, and number of rooms [51]. When combined with downstream exposure models, this approach can then effectively simulate the population with a high level of resolution.

## 5. Conclusions

RPGen creates synthetic populations of individuals with consistent demographic, residential, and physiological characteristics. Output variables can be combined with a wide range of user-developed chemical exposure scenarios to estimate intraindividual exposure in a desired population. While RPGen has been developed initially and applied for use in probabilistic models of consumer product exposure, the tool can be applied to consider a range of environmental health questions. By creating profiles and characteristics that are primary determinants of exposure, RPGen enables the data-driven development of hypotheses related to populations and groups who may be vulnerable to chemical exposures.

Increasingly, the field of exposure science is shifting from a field of observation to a field of prediction [52] and exposure models are quickly evolving to evaluate the complex exposure scenarios presented by commerce [26,49,53]). The aim of RPGen is to inform variability in probabilistic models of exposure by creating a sample population. The benefits are twofold: first, chemical exposure predictions are informed by the correlated physiological, demographic, and housing variables within RPGen. Grouping of these variables into profiles improve the exposure estimates of individuals. Secondary benefit comes from the same variables: age, ethnicity, income, and location allow for comparison with dose outcomes to determine high-risk subpopulations and communities. By using a population that represents the US in exposure models, RPGen better characterize exposure, and in turn, provides demographics to inform policy decisions.

## Figures and Tables

**Table 1:** User inputs to RPGen.

Variable	Class	Input Parameters	Default
Run.name	Character	Name of output folder containing pophouse.csv	User input required
Num.persons	Numeric	Number of individuals (rows) in output	User input required
Min.age	Numeric	Number from 0 to 99	0
Max.age	Numeric	Number from 0 to 99	99
Gender	Character	M = Male F = Female	MF
Ethnicity	Comma separated characters	N = non-Hispanic, M = Mexican-American, O = Other	NMO
Regions	Comma separated numerics	1 = West, 2 = Mid-West, 3 = South, 4 = North West	1234
Race	Comma separated numerics	W = White, B = African American, N = Native American, A = Asian American, P = Pacific Islander, O = Other or Mixed	WBNAPO
States	Comma seperated numerics	FIPS Codes in RPGen User Guide (Available in GitHub repository)	All contiguous US Codes within specified regions.

**Table 2:** Data sets used in this version of the RPGen. Note: NHANES data derived from EPA’s httk.

Survey	Source	Date of Data Collection	Number of Records
Residential Energy Consumption Survey (RECS)	US Energy Information Association: https://www.eia.gov/consumption/residential/data/2015/	2015	5686
American Housing Survey (AHS)	US Census Bureau: https://www.census.gov/programs-surveys/ahs/data.html	2017	57,972
Public Use Microdata Survey (PUMS)	US Census Bureau: https://www.census.gov/programs-surveys/acs/data/pums.html	2014–2018	15,094,428
National Health and Nutrition Examination Survey (NHANES)	US CDC: https://wwwn.cdc.gov/nchs/nhanes/Default.aspx	2007–2008, 2009–2010, 2011–2012	24,546

**Table 3:** Assembly of 288 possible household bins.

Setting	Region	House Type	Family Category	Income Category
Urban	Northeast	Stand Alone	1 Adult, 0 Children	1
Rural	Midwest	Multi Structure	2+ Adults, 0 Children	2
	South	Other	1 Adult, 1+ Children	3
	West		2+ Adults, 1+ Children	

## Data Availability

All code, data, and documentation for RPGen is available at: https://github.com/HumanExposure/RPGen; Last accessed: 11 October 2021.

## Supplementary Materials

The following are available online at https://www.mdpi.com/article/10.3390/toxics9110303/s1, Figure S1: Four Chemicals not used as principal case example for application of RPGen: Benzyl Butyl Pthalate, Dibutyl Pthalate, Diethylene Glycol, Naphthalene daily intakes compared to SHEDS-HT, Table S1: Percentage of Individuals Exposed Across Exposure Model Scenario (Subset), Table S2: Summaries of Daily Intake (mg/kg-bw/day) across Exposure Model Scenario (Subset), Table S3: Counts of Chemicals, Products, Case Example Chemicals, and Ownership Status by PUC ID in CHEM, Table S4: Counts of Products, PUCS, and ‘Homeowner PUCS’ by Chemical in CHEM.4. All code, data, and documentation for RPGen is available at: https://github.com/HumanExposure/RPGen (accessed on 11 October 2021). Metadata: https://edg.epa.gov/metadata/catalog/search/resource/details.page?uuid=%7B91279B3B-AD3D-476A-826A-A0FE0D78AECC%7D (accessed on 11 October 2021).

## References

[1] Dennis K.K., Auerbach S.S., Balshaw D.M., Cui Y., Fallin M.D., Smith M.T., Miller G.W. (2016). The importance of the biological impact of exposure to the concept of the exposome. Environ. Health Perspect..

[2] Wild C.P. (2005). Complementing the genome with an “exposome”: The outstanding challenge of environmental exposure measurement in molecular epidemiology. Cancer Epidemiol. Prev. Biomark..

[3] Wild C.P. (2012). The exposome: From concept to utility. Int. J. Epidemiol..

[4] Rappaport S. (2013). What is the Exposome?.

[5] Vermeulen R., Schymanski E.L., Barabási A.-L., Miller G.W. (2020). The exposome and health: Where chemistry meets biology. Science.

[6] National Resource Council (2009). Science and Decisions: Advancing Risk Assessment.

[7] National Resource Council (2012). Exposure Science in the 21st Century: A Vision and a Strategy.

[8] National Academies of Sciences & Medicine (2017). Using 21st Century Science to Improve Risk-Related Evaluations.

[9] Cushman-Roisin B. (2012). Environmental Transport and Fate.

[10] Vallero D.A. (2019). Air Pollution Calculations: Quantifying Pollutant Formation, Transport, Transformation, Fate and Risks.

[11] Mackay D., Shiu W.-Y., Lee S.C. (2006). Handbook of Physical-Chemical Properties and Environmental Fate for Organic Chemicals.

[12] US Environmental Protection Agency (2017). Guidance for Reporting on the Environmental Fate and Transport of the Stressors of Concern in Problem Formulations. https://www.epa.gov/pesticide-science-and-assessing-pesticide-risks/guidance-reporting-environmental-fate-and-transport.

[13] Hemond H.F., Fechner E.J. (2014). Chemical Fate and Transport in the Environment.

[14] Ali N. (2019). Polycyclic aromatic hydrocarbons (PAHs) in indoor air and dust samples of different Saudi microenvironments; health and carcinogenic risk assessment for the general population. Sci. Total Environ..

[15] Wambaugh J.F., Setzer R.W., Reif D.M., Gangwal S., Mitchell-Blackwood J., Arnot J.A., Cohen-Hubal E. (2013). High-throughput models for exposure-based chemical prioritization in the ExpoCast project. Environ. Sci. Technol..

[16] Csiszar S.A., Meyer D.E., Dionisio K.L., Egeghy P., Isaacs K.K., Price P.S., Vallero D. (2016). Conceptual framework to extend life cycle assessment using near-field human exposure modeling and high-throughput tools for chemicals. Environ. Sci. Technol..

[17] Fantke P., Ernstoff A.S., Huang L., Csiszar S.A., Jolliet O. (2016). Coupled near-field and far-field exposure assessment framework for chemicals in consumer products. Environ. Int..

[18] Csiszar S.A., Ernstoff A.S., Fantke P., Jolliet O. (2017). Stochastic modeling of near-field exposure to parabens in personal care products. J. Expo. Sci. Environ. Epidemiol..

[19] Zhang X., Arnot J.A., Wania F. (2014). Model for screening-level assessment of near-field human exposure to neutral organic chemicals released indoors. Environ. Sci. Technol..

[20] Mitchell J., Arnot J.A., Jolliet O., Georgopoulos P.G., Isukapalli S., Dasgupta S., Hubal E.A.C. (2013). Comparison of modeling approaches to prioritize chemicals based on estimates of exposure and exposure potential. Sci. Total Environ..

[21] Isaacs K.K., Glen W.G., Egeghy P., Goldsmith M.-R., Smith L., Vallero D., Özkaynak H.K. (2014). SHEDS-HT: An integrated probabilistic exposure model for prioritizing exposures to chemicals with near-field and dietary sources. Environ. Sci. Technol..

[22] (2017). Procedures for Chemical Risk Evaluation Under the Amended Toxic Substances Control Act, EPA-HQ-OPPT-2016-0654 C.F.R. https://www.federalregister.gov/documents/2017/07/20/2017-14337/procedures-for-chemical-risk-evaluation-under-the-amended-toxic-substances-control-act.

[23] Fantke P., Charles R., de Alencastro L.F., Friedrich R., Jolliet O. (2011). Plant uptake of pesticides and human health: Dynamic modeling of residues in wheat and ingestion intake. Chemosphere.

[24] Isaacs K.K., Dionisio K., Phillips K., Bevington C., Egeghy P., Price P.S. (2020). Establishing a system of consumer product use categories to support rapid modeling of human exposure. J. Expo. Sci. Environ. Epidemiol..

[25] Sliwinski M.J. (2011). Approaches to modeling intraindividual and interindividual facets of change for developmental research. Handb. Life-Span Dev..

[26] Price P.S., Chaisson C.F. (2005). A conceptual framework for modeling aggregate and cumulative exposures to chemicals. J. Expo. Sci. Environ. Epidemiol..

[27] Finley B., Proctor D., Scott P., Harrington N., Paustenbach D., Price P. (1994). Recommended distributions for exposure factors frequently used in health risk assessment. Risk Anal..

[28] Moya J., Phillips L., Schuda L., Wood P., Diaz A., Lee R., Blood P. (2011). Exposure Factors Handbook.

[29] Huang L., Ernstoff A., Fantke P., Csiszar S.A., Jolliet O. (2017). A review of models for near-field exposure pathways of chemicals in consumer products. Sci. Total Environ..

[30] Dionisio K.L., Frame A.M., Goldsmith M.-R., Wambaugh J.F., Liddell A., Cathey T., Fantke P. (2015). Exploring consumer exposure pathways and patterns of use for chemicals in the environment. Toxicol. Rep..

[31] Washington A.L. (2016). The Interoperability of US Federal Government Information: Interoperability. Big Data: Concepts, Methodologies, Tools, and Applications.

[32] R Core Team (2009). R: A Language and Environment for Statistical Computing.

[33] East A., Price P., Dawson D., Dionisio K., Isaacs K., Hubal E.A.C., Vallero D. The Residential Population Generator (RPGen): Parameterization of Residential, Demographic, and Physiological Data to Model Intraindividual Exposure, Dose, and Risk. https://cfpub.epa.gov/si/si_public_record_Report.cfm?dirEntryId=350496&Lab=CCTE.

[34] US Energy Information Administration (2015). RECS (Residential Energy Consumption Survey). https://www.eia.gov/consumption/residential/reports.php.

[35] US Census Bureau (2019). American Housing Survey Technical Documentatation. https://www.census.gov/programs-surveys/ahs/tech-documentation.html.

[36] US Census Bureau (2020). Public Use Microdata Sample (PUMS). https://www.census.gov/programs-surveys/acs/microdata.html.

[37] Pearce R.G., Setzer R.W., Strope C.L., Wambaugh J.F., Sipes N.S. (2017). Httk: R package for high-throughput toxicokinetics. J. Stat. Softw..

[38] Wambaugh J. High-Throughput Toxicokinetics (HTTK) R Package. Proceedings of the Computational Toxicology Community of Practice Webinar.

[39] Office of Management and Budget (2010). Standards for Metropolitan and Micropolitan Statistical Areas. https://www.federalregister.gov/documents/2010/06/28/2010-15605/2010-standards-for-delineating-metropolitan-and-micropolitan-statistical-areas.

[40] Graham S.E., Langstaff J., Hader J.D., Glen G., Levasseur J. Estimating Fine-Scale Temporal and Spatial Characteristics of SO2 Exposures Using US EPA’s Air Pollutants Exposure (APEX) Model. Proceedings of the ISEE Conference Abstracts.

[41] Ring C.L., Pearce R.G., Setzer R.W., Wetmore B.A., Wambaugh J.F. (2017). Identifying populations sensitive to environmental chemicals by simulating toxicokinetic variability. Environ. Int..

[42] Isaacs K. (2019). SHEDS-HT (Version v0.1.8): GitHub. https://github.com/HumanExposure/SHEDSHTRPackage/releases/tag/v0.1.8.

[43] Breen M.S., Schultz B.D., Sohn M.D., Long T., Langstaff J., Williams R., Smith L. (2014). A review of air exchange rate models for air pollution exposure assessments. J. Expo. Sci. Environ. Epidemiol..

[44] Egeghy P.P., Hubal E.A.C., Tulve N.S., Melnyk L.J., Morgan M.K., Fortmann R.C., Sheldon L.S. (2011). Review of pesticide urinary biomarker measurements from selected US EPA children’s observational exposure studies. Int. J. Environ. Res. Public Health.

[45] US Environmental Protection Agency ExpoBox: A Toolbox for Exposure Assessors 2019. https://www.epa.gov/expobox.

[46] (2014). Congressional Budget Office, The Distribution of Household Income. https://www.cbo.gov/publication/53597.

[47] Sheldon L.S., Cohen Hubal E.A. (2009). Exposure as part of a systems approach for assessing risk. Environ. Health Perspect..

[48] Egeghy P.P., Sheldon L.S., Isaacs K.K., Özkaynak H., Goldsmith M.-R., Wambaugh J.F., Buckley T.J. (2016). Computational exposure science: An emerging discipline to support 21st-century risk assessment. Environ. Health Perspect..

[49] McNally K., Cotton R., Hogg A., Loizou G. (2014). PopGen: A virtual human population generator. Toxicology.

[50] Grefenstette J.J., Brown S.T., Rosenfeld R., DePasse J., Stone N.T., Cooley P.C., Sriram A. (2013). FRED (A Framework for Reconstructing Epidemic Dynamics): An open-source software system for modeling infectious diseases and control strategies using census-based populations. BMC Public Health.

[51] Meng H., Zhang X., Xiao J., Zhang Y., Lin W., Li Z. (2021). A simple physical-activity-based model for managing children’s activities against exposure to air pollutants. J. Environ. Manag..

[52] Teeguarden J.G., Tan Y.-M., Edwards S.W., Leonard J.A., Anderson K.A., Corley R.A., Tanguay R.L. (2016). Completing the Link between Exposure Science and Toxicology for Improved Environmental Health Decision Making: The Aggregate Exposure Pathway Framework.

[53] Cohen Hubal E.A., Richard A., Aylward L., Edwards S., Gallagher J., Goldsmith M.-R., Kavlock R. (2010). Advancing exposure characterization for chemical evaluation and risk assessment. J. Toxicol. Environ. Health Part B.

