# Supplementary material for: The Residential Population Generator (RPGen): Parameterization of Residential, Demographic, and Physiological Data to Model Intraindividual Exposure, Dose, and Risk
Source: Toxics. 2021 Nov 12;9(11):303. doi: 10.3390/toxics9110303 (PMC8625086; doi:10.3390/toxics9110303)
Supplement: Supplementary file 1 [file toxics-09-00303-s001.zip › toxics-1434650 supplementary for proof.pdf]

toxics

Supplementary Materials: The Residential Population Generator (RPGen): Parameterization of Residential, Demographic, and Physiological Data to Model Intraindividual Exposure, Dose, and Risk

Alexander East, Daniel Dawson, Graham Glen, Kristin Isaacs, Kathie Dionisio, Paul S. Price, Elaine A. Cohen Hubal and Daniel A. Vallero

The code and documentation used in this analysis is available at https://github.com/orgs/HumanExposure/repositories. In addition to other modules, this repository contains project files and scripts for RPGen, the Product Use Scheduler (PUS), and Source-to-Dose (S2D), which are the components of the Combined Human Exposure Model (CHEM), in addition to the high-throughput version of the Stochastic Human Exposure Model (SHEDS-HT).

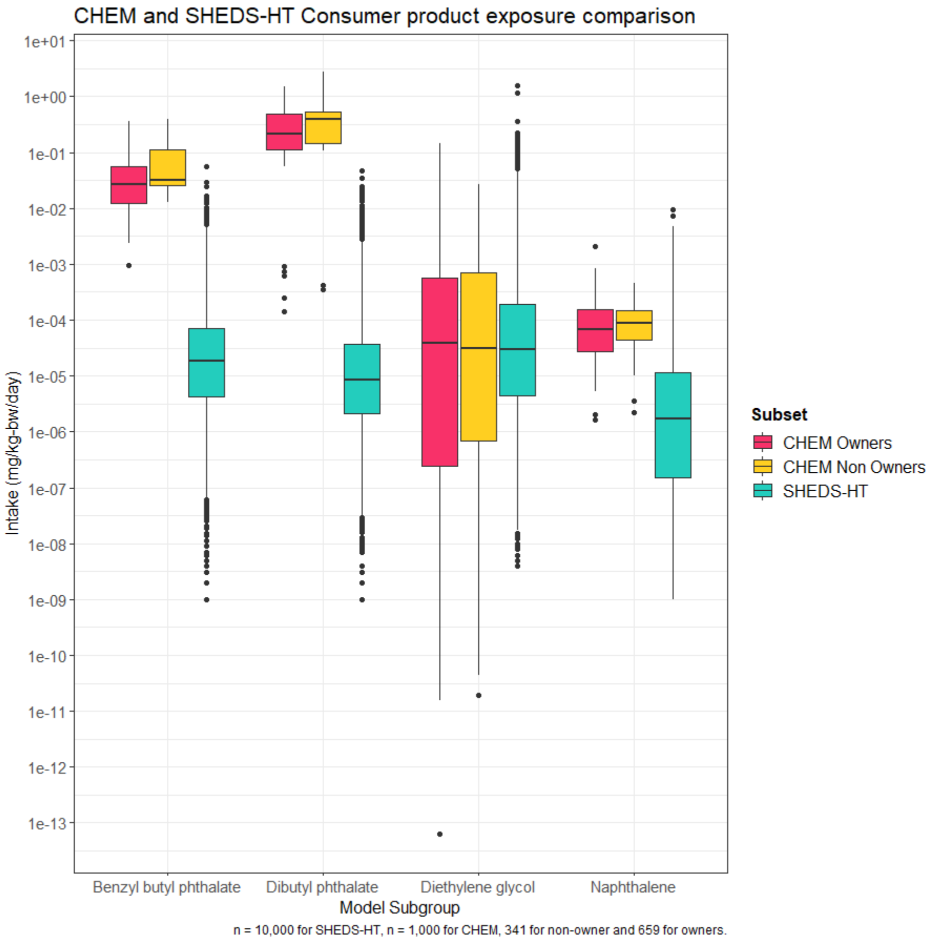

Figure S1. Four Chemicals not used as principal case example for application of RPGen: Benzyl Butyl Pthalate, Dibutyl Pthalate, Diethylene Glycol, Naphthalene daily intakes compared to SHEDS-HT.

In the 1000 individual RPGen population used in this analysis, the four chemicals in Figure 1 were ran through CHEM in addition to toluene. However, these chemicals were

omitted due to relatively infrequent exposure counts in CHEM. Table 1 shows the percentage of individuals exposed to each chemical in Figure 1. Exposure occurred more frequently in SHEDS-HT than CHEM across all subsets. Toluene was selected for the case study given the relatively high exposure frequencies of 69.5%, considering only homeowner product use categories (PUCS), 78.5% among owners, and 36.4% among non-owners. Given the high count of non-exposures, the boxplots in Figure 1 are potentially misleading, as few exposures for napthalene, diethylene glycol, dibutyl pthalate, and benzyl butyl pthalate create the boxplots. "zeros" are not represented, causing distributions orders of magnitude higher than SHEDS-HT. Whereas over 5000 simulated exposures are used to create the SHEDS-HT distributions, as few as 17 are used to create the CHEM distributions. Summary statistics for each model subset output is provided in Table 2 below.

Table S1. Percentage of Individuals Exposed Across Exposure Model Scenario (Subset).

Name	Subset	Exposed	Houses	% Exposed
Diethylene glycol	SHEDS-HT	9998	10000	99.98%
Toluene	SHEDS-HT	9437	10000	94.4%
Napthalene	SHEDS-HT	5512	10000	55.1%
Dibutyl phthalate	SHEDS-HT	5227	10000	52.3%
Benzyl butyl phthalate	SHEDS-HT	5170	10000	51.7%
Toluene	Homeowner PUCS Only	458	659	69.5%
Benzyl butyl phthalate	Homeowner PUCS Only	17	659	2.6%
Naphthalene	Homeowner PUCS Only	17	659	2.6%
Diethylene glycol	Homeowner PUCS Only	16	659	2.4%
Dibutyl phthalate	Homeowner PUCS Only	1	659	0.2%
Toluene	CHEM Owners	517	659	78.5%
Diethylene glycol	CHEM Owners	266	659	40.4%
Naphthalene	CHEM Owners	83	659	12.6%
Dibutyl phthalate	CHEM Owners	54	659	8.2%
Benzyl butyl phthalate	CHEM Owners	32	659	4.9%
Diethylene glycol	CHEM Non Owners	124	341	36.4%
Toluene	CHEM Non Owners	102	341	29.9%
Naphthalene	CHEM Non Owners	40	341	11.7%
Dibutyl phthalate	CHEM Non Owners	28	341	8.2%
Benzyl butyl phthalate	CHEM Non Owners	17	341	5.0%

Table S2. Summaries of Daily Intake (mg/kg-bw/day) across Exposure Model Scenario (Subset).

Name	Subset	Mean	Median	95th Percentile
Benzyl butyl phthalate	SHEDS-HT	0.0000922	0.000000331	0.000264
Dibutyl phthalate	SHEDS-HT	0.000103	0.000000224	0.000166
Diethylene glycol	SHEDS-HT	0.00138	0.0000303	0.00327
Napthalene	SHEDS-HT	0.0000191	7.5E-09	0.0000568
Toluene	SHEDS-HT	0.0233	0.000087	0.0147
Dibutyl phthalate	Homeowner PUCS Only	0.000144	0	0
Benzyl butyl phthalate	Homeowner PUCS Only	0.000609	0	0
Toluene	Homeowner PUCS Only	0.0626	0.00441	0.353
Diethylene glycol	Homeowner PUCS Only	0.000384	0	0
Naphthalene	Homeowner PUCS Only	0.00000829	0	0
Dibutyl phthalate	CHEM Owners	0.0265	0	0.152
Benzyl butyl phthalate	CHEM Owners	0.00221	0	0
Toluene	CHEM Owners	0.0671	0.00585	0.375
Diethylene glycol	CHEM Owners	0.00104	0	0.00337
Naphthalene	CHEM Owners	0.0000169	0	0.0000842
Dibutyl phthalate	CHEM Non-owners	0.0439	0	0.244

Benzyl butyl phthalate	CHEM Non-owners	0.00498	0	0
Toluene	CHEM Non-owners	0.00406	0	0.0139
Diethylene glycol	CHEM Non-owners	0.000457	0	0.00182
Naphthalene	CHEM Non-owners	0.000013	0	0.0000999

Toluene was selected given the prevalence of the chemical in products associated with home ownership. In CHEM, the likelihood of exposure is a function of the probability of being assigned a product use category times the probability of a product being selected which contains the chemical of interest. Therefore, CHEM is best used for chemicals occuring in many products and product use categories. A composition of Product Use Category Identifiers (PUCIDs) with regards to case example chemicals and home ownership classification is presented in Table 3 below. Table 4 provides counts of the number of PUCS and products containing each chemical.

Table S3. Counts of Chemicals, Products, Case Example Chemicals, and Ownership Status by PUCID in CHEM.

PUCID	Total Chemicals	Total Products	Description	Case Example Chemicals	Homeownership
CP.0100.010.099.F	139	128	air freshener, air freshener NOC	Toluene	No
VE.0100.030.099.F	56	75	auto body work, detailing NOC	Toluene	No
AC.0500.010.099.F	41	44	general arts and crafts supplies, arts and crafts adhesive NOC	Toluene	No
VE.0100.030.050.F	20	20	auto body work, detailing spray	Toluene	No
HM.1400.020.099.F	15	6	paint/stain and related products, paint cleaner NOC	Toluene	Yes
HM.1400.070.099.F	15	15	paint/stain and related products, stripper NOC	Toluene	Yes
CP.1600.010.099.F	14	20	shoes, shoe polish or protectant NOC	Toluene	No
HM.0600.010.050.F	13	6	corrosion protection, corrosion protection spray	Toluene	Yes
VE.0100.010.050.F	12	15	auto body work, auto paint spray	Toluene	No
VE.0500.020.050.F	11	6	engine maintenance, auto fluids and additives spray	Toluene	No
HM.1400.040.099.F	10	7	paint/stain and related products, paint thinner NOC	Toluene	Yes
AC.0500.030.050.F	10	2	general arts and crafts supplies, arts and crafts finish spray	Toluene	No
AC.0600.010.099.F	7	5	gun cleaner, gun cleaner NOC	Toluene	No
AC.0500.030.099.F	6	3	general arts and crafts supplies, arts	Toluene	No

			and crafts finish NOC		
HM.1400.050.050.F	5	4	paint/stain and related products, primer spray	Toluene	Yes
HM.0100.030.099.F	3	3	adhesives and adhesive removers, wood adhesive NOC	Toluene	Yes
HM.0800.010.099.F	28	14	finish, finish NOC	Naphthalene, Toluene	Yes
VE.0400.040.099.F	10	16	car surface treatment, degreaser NOC	Naphthalene, Toluene	No
VE.0500.050.099.F	7	44	engine maintenance, motor oil NOC	Naphthalene, Toluene	No
VE.0500.020.099.F	59	68	engine maintenance, auto fluids and additives NOC	Diethylene glycol, Naphthalene, Toluene	No
LY.0200.010.099.F	26	20	grill/camping fuel, grill/camping fuel NOC	Diethylene glycol, Naphthalene, Toluene	Yes
VE.0200.040.099.F	22	3	boat care and maintenance, boat engine fluids NOC	Diethylene glycol, Naphthalene, Toluene	Yes
AC.0700.020.099.F	69	88	home office, pens and markers NOC	Diethylene glycol	No
CP.1200.060.099.F	67	194	laundry and fabric treatment, laundry detergent NOC	Diethylene glycol	No
CP.1200.060.052.F	44	116	laundry and fabric treatment, laundry detergent liquid	Diethylene glycol	No
CP.1100.010.099.F	32	26	lamp oil/lighter fluid, lamp oil/lighter fluid NOC	Diethylene glycol	No
HM.1400.010.041.F	14	85	paint/stain and related products, paint interior	Diethylene glycol	Yes
VE.0500.010.099.F	7	21	engine maintenance, antifreeze NOC	Diethylene glycol	No
CP.1200.030.099.F	5	12	laundry and fabric treatment, fabric deodorizer NOC	Diethylene glycol	No
HM.0100.020.099.F	58	65	adhesives and adhesive removers, multipurpose adhesive NOC	Dibutyl phthalate, Toluene	Yes
HM.1400.010.050.F	39	126	paint/stain and related products, paint spray	Dibutyl phthalate, Toluene	Yes
VE.0100.020.099.F	30	16	auto body work, body repair NOC	Dibutyl phthalate, Toluene	No
AC.0500.010.050.F	15	8	general arts and crafts supplies, arts and crafts adhesive spray	Dibutyl phthalate, Toluene	No

CP.0300.040.099.F	7	7	carpet and floor, floor polish NOC	Dibutyl phthalate	No
HM.0400.010.099.F	40	31	caulk/sealant, caulk/sealant NOC	Benzyl butyl phthalate, Toluene	Yes
HM.0800.010.050.F	13	5	finish, finish spray	Benzyl butyl phthalate, Toluene	Yes
HM.2000.040.050.F	6	1	surface sealers, surface sealer spray	Benzyl butyl phthalate, Toluene	Yes
AC.0500.040.099.F	52	93	general arts and crafts supplies, arts and crafts paint NOC	Benzyl butyl phthalate, Naphthalene, Toluene	No
VE.0100.010.099.F	22	35	auto body work, auto paint NOC	Benzyl butyl phthalate, Naphthalene, Toluene	No
HM.1400.050.099.F	16	8	paint/stain and related products, primer NOC	Benzyl butyl phthalate, Naphthalene, Toluene	Yes
EL.0300.050.099.F	31	169	computers and accessories/supplies, printer ink NOC	Benzyl butyl phthalate, Diethylene glycol, Toluene	No
HM.1400.010.040.F	23	57	paint/stain and related products, paint exterior	Benzyl butyl phthalate, Diethylene glycol, Toluene	Yes
HM.1400.010.099.F	30	87	paint/stain and related products, paint NOC	Benzyl butyl phthalate, Diethylene glycol, Naphthalene, Toluene	Yes
HM.2100.010.099.F	6	2	tiling, grout sealer NOC	Benzyl butyl phthalate	Yes
HM.1500.020.099.F	5	3	patch and repair, putty or filler NOC	Benzyl butyl phthalate	Yes

Table S4. Counts of Products, PUCS, and 'Homeowner PUCS' by Chemical in CHEM.4

Name	Products	PUCS	Homeowner PUCS	PUC Names	Uses
Toluene	229	35	17	HM.0100.020.099.F, HM.0100.030.099.F, HM.0400.010.099.F, HM.0600.010.050.F, HM.0800.010.050.F, HM.0800.010.099.F, HM.1400.010.040.F, HM.1400.010.050.F, HM.1400.010.099.F, HM.1400.020.099.F, HM.1400.040.099.F, HM.1400.050.050.F, HM.1400.050.099.F, HM.1400.070.099.F, HM.2000.040.050.F, LY.0200.010.099.F, VE.0200.040.099.F	paint/stain and related products, primer, general arts and crafts supplies, arts and crafts finish, general arts and crafts supplies, arts and crafts paint, auto body work, auto paint, adhesives and adhesive removers, multipurpose adhesive, paint/stain and related products , paint spray, corrosion protection , corrosion protection spray, engine maintenance, auto fluids

and additives, paint/stain and related products, paint NOC, auto body work, body repair, shoes , shoe polish or protectant NOC, grill/camping fuel , grill/camping fuel NOC, auto body work , auto paint spray, finish , finish NOC, engine maintenance , auto fluids and additives spray, engine maintenance , motor oil NOC, general arts and crafts supplies , arts and crafts adhesive spray, surface sealers , surface sealer spray, car surface treatment , degreaser NOC, paint/stain and related products , stripper NOC, paint/stain and related products , paint cleaner NOC, finish , finish spray, paint/stain and related products , primer spray, general arts and crafts supplies , arts and crafts adhesive NOC, paint/stain and related products , paint thinner NOC, auto body work , detailing NOC, paint/stain and related products , paint exterior, general arts and crafts supplies , arts and crafts finish spray, adhesives and adhesive removers , wood adhesive NOC, boat care and maintenance , boat engine fluids NOC, auto body work , detailing spray, gun cleaner , gun cleaner NOC, computers and accessories/supplies ,

					printer ink NOC, air freshener , air freshener NOC, caulk/sealant , caulk/sealant NOC
Benzyl butyl phthalate	27	11	8	HM.0400.010.099.F, HM.0800.010.050.F, HM.1400.010.040.F, HM.1400.010.099.F, HM.1400.050.099.F, HM.1500.020.099.F, HM.2000.040.050.F, HM.2100.010.099.F	general arts and crafts supplies, arts and crafts paint NOC, finish , finish spray, auto body work , auto paint NOC, paint/stain and related products , primer NOC, patch and repair , putty or filler NOC, caulk/sealant , caulk/sealant NOC, surface sealers , surface sealer spray, tiling , grout sealer NOC, paint/stain and related products , paint exterior, computers and accessories/supplies , printer ink NOC, paint/stain and related products , paint NOC
Diethylene glycol	211	13	5	HM.1400.010.040.F, HM.1400.010.041.F, HM.1400.010.099.F, LY.0200.010.099.F, VE.0200.040.099.F	computers and accessories/supplies , printer ink NOC, laundry and fabric treatment , laundry detergent liquid, laundry and fabric treatment , laundry detergent NOC, paint/stain and related products , paint NOC, grill/camping fuel , grill/camping fuel NOC, paint/stain and related products , paint interior, engine maintenance , auto fluids and additives NOC, engine maintenance , antifreeze NOC, home office , pens and markers NOC, paint/stain and related products , paint exterior, lamp oil/lighter fluid , lamp oil/lighter fluid NOC, boat care and

					maintenance , boat engine fluids NOC, laundry and fabric treatment , fabric deodorizer NOC
Naphthalene	46	10	5	HM.0800.010.099.F, HM.1400.010.099.F, HM.1400.050.099.F, LY.0200.010.099.F, VE.0200.040.099.F	general arts and crafts supplies, arts and crafts paint NOC, engine maintenance, auto fluids and additives NOC, auto body work , auto paint NOC, paint/stain and related products , paint NOC, boat care and maintenance , boat engine fluids NOC, car surface treatment , degreaser NOC, grill/camping fuel , grill/camping fuel NOC, finish , finish NOC, paint/stain and related products , primer NOC, engine maintenance , motor oil NOC
Dibutyl phthalate	6	5	2	HM.0100.020.099.F, HM.1400.010.050.F	carpet and floor, floor polish NOC, auto body work, body repair NOC, adhesives and adhesive removers , multipurpose adhesive NOC, paint/stain and related products , paint spray, general arts and crafts supplies , arts and crafts adhesive spray
